# Patterns of pontine strokes mimicking Bell’s palsy

**DOI:** 10.1186/s12883-019-1440-1

**Published:** 2019-08-27

**Authors:** Young Gi Min, Keun-Hwa Jung

**Affiliations:** Department of Neurology, Seoul National University Hospital, 101 Daehangno, Jongno-gu, Seoul, 110-744 South Korea

**Keywords:** Peripheral-type facial paralysis, Ophthalmoplegia, Pons, Stroke, Stroke, lacunar

## Abstract

**Background:**

Peripheral-type facial palsy very rarely arises from pontine stroke. We attempted to identify unique clinico-radiologic patterns associated with this condition.

**Case presentation:**

Patients with pontine tegmentum stroke and acute onset of peripheral-type facial weakness were reviewed from the acute stroke registry of a tertiary hospital. The clinico-radiologic patterns of 10 patients were classified into one of three types based on the respective stroke mechanism. Type A (*n* = 5) was characterized by relatively diverse clinical presentations and larger, multiple infarctions resulting from large-artery atherosclerosis. Three cases with small lacunar infarcts were classified to type B (small vessel occlusion), and they showed only limited symptoms including horizontal gaze disturbance and facial paralysis. The two hemorrhagic cases (type C) presented with a focal pontine hemorrhage, likely due to a cavernous hemangioma.

**Conclusions:**

Peripheral-type facial palsy often occurs in pontine stroke with specific patterns. Type recognition helps to determine the underlying mechanism and the appropriate clinical approach. In particular, focal pontine tegmental infarctions showing stereotypic combinations of ophthalmoplegia and peripheral-type facial weakness (type B) might be recognized as a new type of lacunar syndrome.

## Background

Facial weakness frequently occurs along with ipsilateral hemiparesis in pure motor lacunar syndrome. Forehead sparing usually occurs in these cases, indicating supranuclear pathology. We recently encountered a patient with peripheral-type facial weakness as the result of scattered embolic infarctions in the vertebrobasilar territory, which is very uncommon in clinical practice. Hence, we reviewed patients with pontine stroke characterized by peripheral-type facial weakness and suggest three distinct features of stroke that trigger facial weakness of the lower motor neuron type.

## Case presentation

### Representative case

**Fig. 1:**
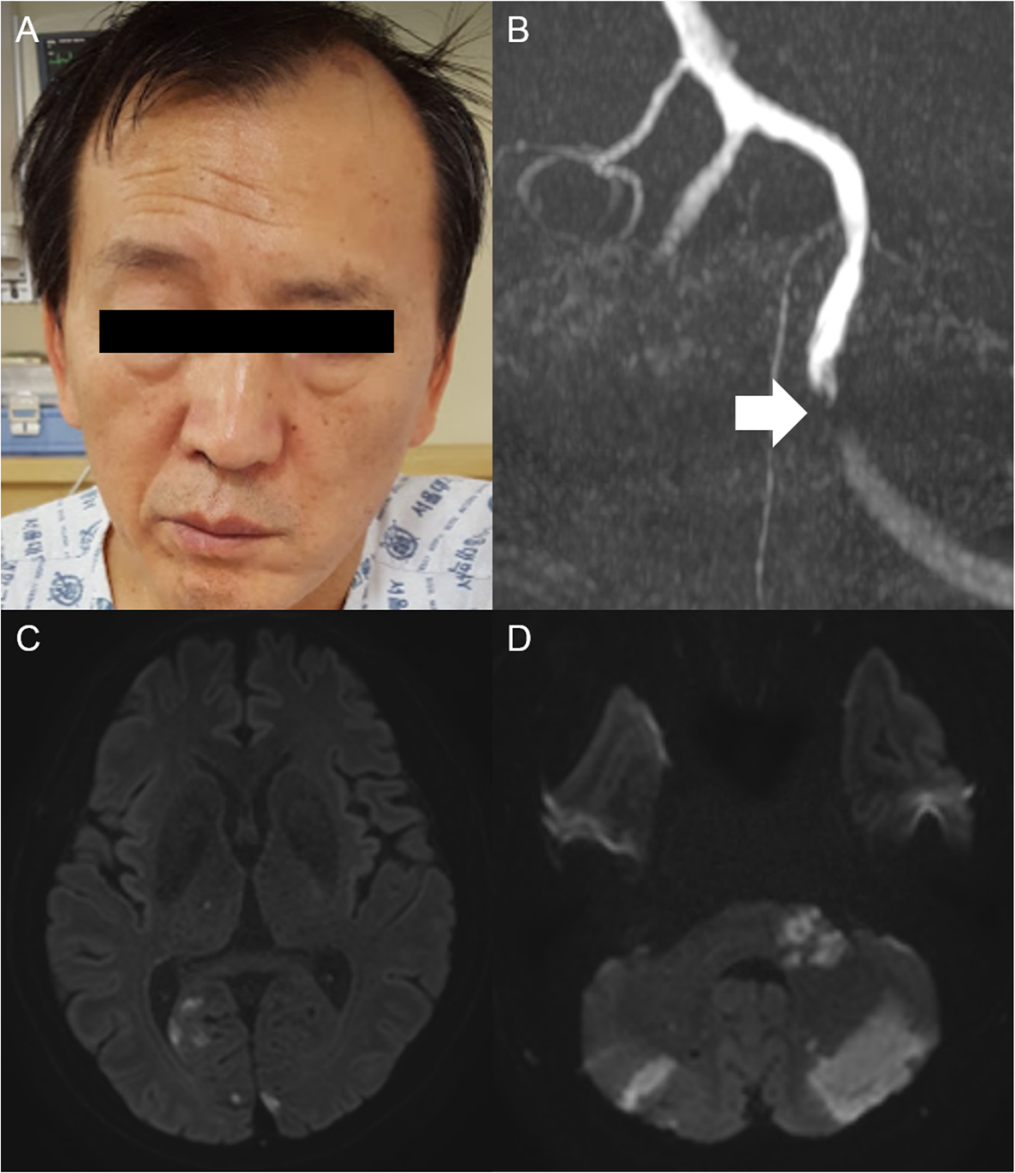
Findings on physical exam and magnetic resonance imaging of the representative case. **a** A 58-year-old man complained of sudden left peripheral-type facial nerve palsy. **b** MRA - A focal occlusion of the left vertebral artery; (**c**, **d**) DWI - Multiple scattered infarctions in bilateral vertebrobasilar territory

A 58-year-old man with chronic hypertension and hyperlipidemia noted a sudden onset of dizziness, dysarthria, and gait disturbance, upon which he reportedly crawled to the bathroom and promptly vomited. Subsequently, he noted left facial weakness. Neurological examination revealed left peripheral-type facial weakness, characterized by a loss of the forehead crease and lowering of the eyebrow (Fig. 1a). Taste, hearing, and inner ear canal sensation were unimpaired. There was no limb weakness, but the ipsilateral limbs were ataxic. Diffusion-weighted image revealed scattered infarctions distributed in the vertebrobasilar system (Fig. 1c and d). Magnetic resonance angiography (MRA) revealed a focal occlusion of the left vertebral artery (Fig. 1b). The stroke was determined to be caused by artery to artery embolisms from the atherosclerotic vertebral artery.

### Clinico-radiologic characteristics of index cases

**Fig. 2:**
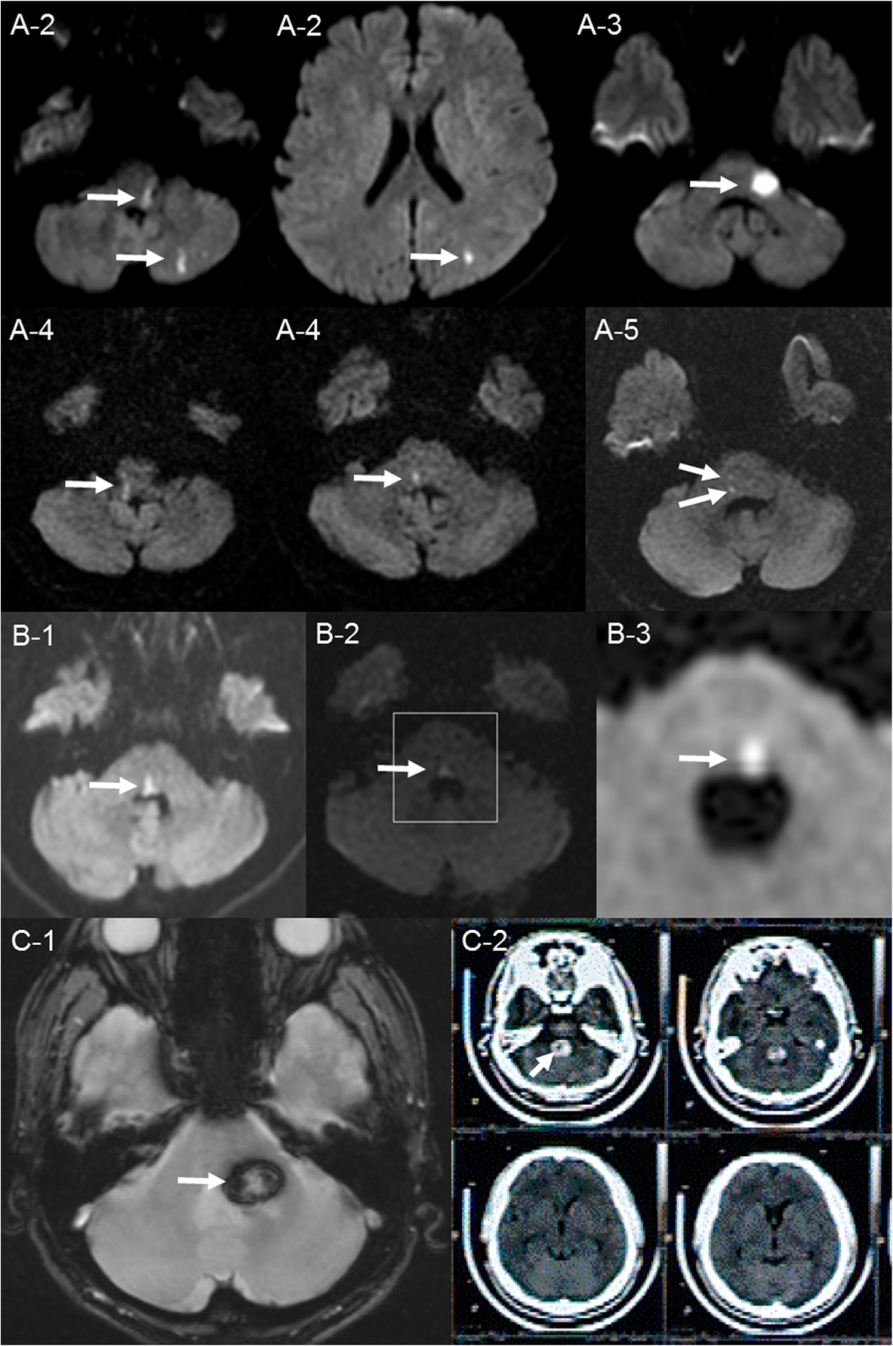
Radiologic findings of nine cases. Radiologic findings of nine cases, except the representative case (Case A-1). (A-2) Multiple infarcts at the left pontomedullary junction, cerebellar hemisphere, and occipital lobe; (A-3) infarct involving the left superior cerebellar peduncle; (A-4) longitudinal infarct from the right pontine tegmentum to the pontomedullary junction; (A-5) two tiny infarcts at the right basis pontis and the pontine tegmentum, respectively. (B-1) Focal infarct at the midline extending to the right pontine tegmentum adjacent to the 4th ventricle; (B-2) focal infarct at the midline pontine tegmentum adjacent to the 4th ventricle; (B-3) focal infarct at the left pontine tegmentum adjacent to the 4th ventricle. (C-1) Pontine hemorrhage presumably due to cavernous malformation at the left middle cerebellar peduncle; (C-2) pontine hemorrhage due to cavernous malformation predominantly involving the ventral aspect of the 4th ventricle

The patients with stroke involving pontine area were collected from the acute stroke registry of a tertiary hospital between 2005 and 2018. With a thorough review of their medical records, patients with evident peripheral-type facial paralysis of a clear onset affecting both the upper and lower face were selected, while those with insufficient alertness or awareness to properly cooperate with the examination were excluded. Eventually, we identified 10 patients who manifested a clear acute onset of peripheral-type facial palsy attributed to pontine stroke and investigated their clinical and radiologic characteristics. Radiologic findings, laboratory investigations, such as blood profiles (Hb A1c, lipid panel), echocardiography, and Holter monitor were reviewed. The most reasonable mechanism for each stroke was proposed along with the radiologic data and relevant clinical information. Cases of stroke were categorized into one of the following three types according to the TOAST classification system: large-artery atherosclerosis (type A), small vessel occlusion (type B), or hemorrhagic (type C) [1]. Table 1 summarizes the clinico-radiologic characteristics of 10 cases. Figure 2 shows their important radiologic findings, except the representative case (Case A-1) which is already described in Fig. 1.

**Table 1:** Clinico-radiologic characteristics of 10 cases

No	S/A	Chief complaint	Neurological exam	Lesion location	Mechanism
A-1	M/58	Dizziness, unsteady gait, dysarthria	Left facial palsy, left limb ataxia	Left middle cerebellar peduncle, bilateral cerebellar hemispheres, bilateral occipital lobes	Large-artery atherosclerosis
A-2	M/51	Dizziness, transient left side weakness	Left facial palsy, GEN, no objective motor weakness	Left pontomedullary junction, left cerebellar hemisphere, left occipital lobe	Large-artery atherosclerosis
A-3	M/57	Left tinnitus, left hearing loss, unsteady gait	Left facial palsy, left limb ataxia, left positive HIT, left SNHL	Left superior cerebellar peduncle	Large-artery atherosclerosis
A-4	M/68	Dizziness	Right facial palsy, GEN	From right pontine tegmentum to pontomedullary junction	Large-artery atherosclerosis
A-5	M/74	Dizziness, diplopia	Right facial palsy, no objective EOM limit	Right basis pontis, right pontine tegmentum	Large-artery atherosclerosis
B-1	M/46	Dizziness, diplopia	Right eight-and-a-half syndrome [7]	Right pontine tegmentum	Small vessel occlusion
B-2	M/55	Dizziness, dysarthria	Sixteen syndrome [8]	Midline pontine tegmentum	Small vessel occlusion
B-3	M/62	Dizziness, diplopia	Left eight-and-a-half syndrome [7]	Left pontine tegmentum	Small vessel occlusion
C-1	M/49	Dizziness, diplopia, unsteady gait, left tinnitus, left hearing loss	Left facial palsy, left limb ataxia, left SNHL, Brun’s nystagmus	Left middle cerebellar peduncle	Hemorrhagic
C-2	M/40	Neck pain, diplopia	Left facial palsy, left horizontal gaze palsy	Midline pontine tegmentum	Hemorrhagic

Abbreviations: *S/A* sex/age, *M* male, *GEN* gaze evoked nystagmus, *HIT* head impulse test, *SNHL* sensorineural hearing loss, *EOM* extraocular movement

Five cases of patients (Cases A-1 to A-5) with diverse clinical presentations that included audiovestibular dysfunction, gaze evoked nystagmus (GEN), and cerebellar ataxia, were classified as type A. Lesions of type A were mainly localized in the lateral pons with occasional extension into the ipsilateral medulla, cerebellar hemisphere, or occipital lobes. In contrast, three cases of patients showing symptoms and signs limited to binocular diplopia or gaze disturbance along with facial weakness were classified as type B (Cases B-1 to B-3). The responsible lesions were focally located in the paramedian pons adjacent to the floor of the fourth ventricle. The remaining two cases presented with a single focal pontine hemorrhage at a relatively young age (at 40 and 49 years, respectively) without clear documentation of chronic hypertension. Considering the atypical involvement of the dorsal pontine tegmentum, the absence of hypertension, and the unremarkable MRA findings, these cases were categorized as type C (hemorrhagic), likely due to the presence of cavernous hemangiomas.

## Discussion and conclusions

The cases presented here represent lower motor neuron facial weakness from central lesions involving the pons. Although a classic Foville syndrome with ipsilateral peripheral-type facial palsy accompanying contralateral hemiparesis with horizontal ocular disturbance from a single pontine lesion is frequently mentioned in textbooks, we have yet to see a clear-cut case in an alert patient with an ischemic stroke [2, 3].

Our case series highlights two major patterns of pontine infarcts that resulted in peripheral-type facial weakness. The lateral pontine lesion is mainly found in type A, which involves the pontine circumferential vessel or anterior inferior cerebellar artery territory, and sometimes shows multi-vessel involvement (Cases A-1 and A-2). The relevant mechanism was determined to be artery to artery embolism (Cases A-1 and A-2) or branch atheromatous disease (Cases A-3 to A-5) [4]. These lesions likely interrupt the distal facial nerve fascicles destined to exit in the cerebellopontine angle after looping around the ipsilateral abducens nucleus.

Type B is clinically characterized by relatively restricted clinical syndromes, including peripheral-type facial involvement and/or horizontal ocular disturbance. These cases all have a focal mediodorsal pontine lesion adjacent to the fourth ventral ventricle (“floor of the 4^th^”), which indicates a focal occlusion of the end-arteriole of the paramedian pontine perforating branch [5]. Ocular signs with lower motor neuron facial weakness have been given several numerical eponyms after Miller-Fisher’s original description of one-and-a-half syndrome [6]. The additional presence of ipsilateral peripheral facial nerve involvement has been described as an eight-and-a-half syndrome (Cases B-1 and B-3), and the bilateral horizontal gaze limitation associated with bilateral facial nerve involvement is described as “16” syndrome (Case B-2) [7, 8]. These stereotypic combinations should be recognized as a new type of lacunar syndrome. Taking into consideration the single small-sized infarction, absence of luminal irregularity on MRA, and observation of clear wall demarcation on relevant axial T2 images, small vessel occlusion was presumed to be the pathogenetic mechanism of the disease in cases B-1 to B-3. However, to further clarify the mechanism, more advanced imaging techniques, such as high-resolution MRA, may be required [9].

Aside from the ischemic mechanism, hemorrhagic stroke could be considered as a potential cause of peripheral-type facial palsy. In this case, the lesion is supposed to be small and specifically located around the VII nucleus and fascicle as our cases go.

In conclusion, recognition of the manifestations of the pontine strokes outlined above may help physicians to elicit the mechanism of stroke and underlying vascular risk factors.

## Data Availability

The datasets used and/or analyzed during the current study are available from the corresponding author on reasonable request.

## Abbreviations

GEN: gaze evoked nystagmus
MRA: Magnetic resonance angiograph

## References

[1] Adams HP, Bendixen BH, Kappelle LJ, Biller J, Love BB, Gordon DL (1993). Classification of subtype of acute ischemic stroke. Definitions for use in a multicenter clinical trial. TOAST. Trial of ORG 10172 in acute stroke treatment. Stroke..

[2] Silverman IE, Liu GT, Volpe NJ, Galetta SL (1995). The crossed paralyses. The original brain-stem syndromes of Millard-Gubler, Foville, weber, and Raymond-Cestan. Arch Neurol.

[3] Borgna C, Fiengo L, Ture U (2012). Achille Louis Foville’s atlas of brain anatomy and the Defoville syndrome. Neurosurgery..

[4] Petrone L, Nannoni S, Del Bene A, Palumbo V, Inzitari D (2016). Branch atheromatous disease: a clinically meaningful, yet unproven concept. Cerebrovasc Dis.

[5] Caplan LR (2015). Lacunar infarction and small vessel disease: pathology and pathophysiology. J Stroke.

[6] Fisher CM (1967). Some neuro-ophthalmological observations. J Neurol Neurosurg Psychiatry.

[7] Eggenberger ER (1998). Eight-and-a-half syndrome: one-and-a-half syndrome plus cranial nerve VII palsy. J Neuroophthalmol.

[8] Connors R, Ngan V, Howard J (2013). A case of complete lateral gaze paralysis and facial diplegia: the 16 syndrome. J Neuroophthalmol.

[9] Xia C, Chen HS, Wu SW, Xu WH (2017). Etiology of isolated pontine infarctions: a study based on high-resolution MRI and brain small vessel disease scores. BMC Neurol.

